# Modern Environmental Health Hazards in Africa: Additional Comments

**DOI:** 10.1289/ehp.0900669

**Published:** 2009-07

**Authors:** José G. Dórea

**Affiliations:** Faculty of Health Sciences, Universidade de Brasilia, Brasilia, Brazil, E-mail: dorea@rudah.com.br

I commend *Environment Health Perspectives* for publishing the work of Nweke and Sanders (2009); this significant contribution brought to light interesting aspects of environmental health hazards on the African continent that can be universalized for scientifically unrepresented less developed countries and regions of the planet. In this dimension, existing studies [World Health Organization (WHO) 1989] of that continent compel us to share additional results of studies of heavy metals (mercury, lead, and cadmium) related to early exposure in children. I would like to address the WHO (1989) study of breast milk concentrations used to monitor mother–infant contamination in selected African countries (Nigeria and Zaire). I would also like to point out an often neglected but universal source of Hg exposure during pregnancy and throughout infancy and childhood—ethylmercury (etHg) in thimerosal-containing vaccines (TCVs).

Concentrations of Hg and Pb in breast milk are important indicators of prenatal exposure, the period when most neurotoxic insults of these elements occur. In a review in which I summarized the WHO (1989) study, I showed that mean Hg concentrations were similar in both Nigeria and Zaire (Dórea 2004), but these concentrations were among the highest reported in that review (Dórea 2004). However, the concentrations of milk Pb were higher than that of milk Hg for both countries; in the case of Pb, mean Pb concentrations in rural Zairians were twice that of urban dwellers. On a molar basis, there was twice as much Pb as Hg in these African countries (Dórea 2004); however, the ratios of Se and Ca concentrations (attenuators of neurotoxicity of Hg and Pb) were quite different between the two countries.

Nweke and Sanders (2009) realized that the earliest stages of neurodevelopment are most vulnerable to the toxic effects of Hg. Therefore, I find the figures of occupational exposure involving mothers to be disturbing; women occupationally exposed to gold processing from amalgam range from 5% of the population in South Africa to 50% in Mali. Also, African women are exposed to Hg in soap and through traditional fish consumption. However, Nweke and Sanders (2009) did not mention that tetanus vaccines are used in countries following WHO recommendations to control or eradicate maternal and neonatal tetanus. These vaccines are preserved with thimerosal. In any part of the developing world where TCVs are in widespread use, a newborn is exposed to high concentrations of etHg depending on the child’s weight and vaccine brand (Dórea and Marques 2008). Indeed, because the hepatitis-B vaccine is given within hours of birth, Hg concentrations can reach extremely high levels of acute exposure, depending on birth weight and vaccine manufacturer (Dórea and Marques 2008). These exposures are higher than the ones estimated for occupationally exposed mothers working with gold extraction (Dórea 2009). Nweke and Sanders (2009) covered environmental hazards as a result of exposure to hazardous pollutants in tandem with development activities, as well as evidence of their adverse effects on African populations. However, they did not mention this important source of Hg exposure to which the fetus (during pregnancy), infant, or child is exposed.

Some African populations, due to lack of sanitation and hygiene, are more prone to preventable diseases and are, as a result, a target for vaccination campaigns for children’s diseases; additionally, emergency measures may introduce specific vaccines for diseases that are rare (or nonexistent), eradicated, or controlled in other countries. Some of these vaccines, for operational reasons, need thimerosal as a preservative. Currently, because of the low cost, TCVs are routinely used in underdeveloped countries, whereas the European Union, the United States, and other industrialized countries have stopped using them based on the plausibility that TCVs may affect neurodevelopment of young children. These precautionary measures need to reach the great majority of infants and young children around the world (including Africa).

Given the heterogeneous socioeconomic situation of African countries, differences in need for vaccines and the affordability of mass immunization programs are complex and difficult to study. Although I support mass vaccination, it is important to take into account characteristics of the health status of African populations that put groups at risk because of their increased suceptibility to Hg neurotoxicity. As recognized by Nweke and Sanders (2009), Africa’s environmental health issues are complex; the environmental health policies and actions of the continent should be comprehensive, holistic, and population specific in the identification, recognition, and management of environmental health hazards. Additionally, the transition to addressing modern environmental health hazards in Africa is also occurring in other parts of the world that have a similar combination of pre-industrial and industrial era environmental health issues combined with the disease burden of children.

## Editor’s note

In accordance with journal policy, Nweke and Sanders were asked whether they wanted to respond to this letter, but they chose not to do so.
